# Latitude in sample handling and storage for infant faecal microbiota studies: the elephant in the room?

**DOI:** 10.1186/s40168-016-0186-x

**Published:** 2016-07-30

**Authors:** Alexander G. Shaw, Kathleen Sim, Elizabeth Powell, Emma Cornwell, Teresa Cramer, Zoë E. McClure, Ming-Shi Li, J. Simon Kroll

**Affiliations:** 1 Department of Medicine, Section of Paediatrics, Imperial College London, London, UK; 2 Royal Holloway, Surrey, UK

**Keywords:** Microbiota, Storage, Frozen, Stability, Mail, Weight, Faeces

## Abstract

**Background:**

In this manuscript, we investigate the “stones best left unturned” of sample storage and preparation and their implications for the next-generation sequencing of infant faecal microbial communities by the 16S ribosomal ribonucleic acid (rRNA) gene.

We present a number of experiments that investigate the potential effects of often overlooked methodology factors, establishing a “normal” degree of variation expected between replica sequenced samples. Sources of excess variation are then identified, as measured by observation of alpha diversity, taxonomic group counts and beta diversity magnitudes between microbial communities.

**Results:**

Extraction of DNA from samples on different dates, by different people and even using varied sample weights results in little significant difference in downstream sequencing data. A key assumption in many studies is the stability of samples stored long term at −80 °C prior to extraction. After 2 years, we see relatively few changes: increased abundances of lactobacilli and bacilli and a reduction in the overall OTU count. Where samples cannot be frozen, we find that storing samples at room temperature does lead to significant changes in the microbial community after 2 days. Mailing of samples during this time period (a common form of sample collection from outpatients for example) does not lead to any additional variation.

**Conclusions:**

Important methodological standards can be drawn from these results; painstakingly created archives of infant faecal samples stored at −80 °C are still largely representative of the original community and varying factors in DNA extraction methodology have comparatively little effect on overall results. Samples taken should ideally be either frozen at −80 °C or extracted within 2 days if stored at room temperature, with mail samples being mailed on the day of collection.

**Electronic supplementary material:**

The online version of this article (doi:10.1186/s40168-016-0186-x) contains supplementary material, which is available to authorized users.

## Background

Developments in massively parallel sequencing technology have transformed our ability to study complex bacterial populations. They offer penetrating insights into human, animal and environmental microbial ecology through the analysis (for example by 16S rRNA gene sequencing) of large collections of samples, often accumulated in the field over an extended period of time. The new technology presents new challenges in the handling of these samples. Samples—which may vary in size from milligrammes to grammes—may be in transit for hours to days under variable ambient conditions before reaching the laboratory, where whilst in some cases, DNA may be extracted at once and held for later analysis; in others, samples may be stored frozen for variable (and sometimes extended) periods before processing. There are concerns about the changes that these environmental factors introduce, different aspects of which have been addressed by various investigators [1–9].

In the course of a study of the developing infant faecal microbiota, extending over 3 years, we have faced many of these issues. Some of our early infant faecal samples have been minute. Whilst some have been collected in hospital, stored quickly at −20 °C and shortly afterwards at −80 °C, others have been collected at the infant’s home by a research nurse and transported back to the laboratory at room temperature (~20 °C) only reaching frozen storage at the end of the day. As the study progressed, a practical solution to the collection of samples from our increasingly dispersed subject population has been for parents to collect them at home and mail them (suitably packaged) via the UK mail system. With no environmental control in transit, these packages arrive in our Institution’s mail room, where the temperature regularly reaches 30 °C. By whatever means the samples reach the laboratory, they are subsequently stored at −80 °C, some for many months, before further processing.

Now we report the results of an investigation of the impact of these different factors on data describing the microbial communities of the samples on the basis of 16S rRNA gene sequence.

## Methods

### Study populations

Data analysed in this study is derived from 103 faecal samples collected from two infant cohorts. The first cohort consists of infants participating in the Neonatal Microbiota (NeoM) study, who were born prematurely (<32 completed weeks of gestation). Faecal samples were collected between the time of the baby’s admission to the NICU at St Mary’s Hospital or Queen Charlotte’s and Chelsea Hospital and discharge. The cohort is fully described in Sim et al. [10]. Samples used here were collected from eight infants aged between 2 and 11 weeks. Such samples represent relatively simple bacterial communities. The second cohort consists of healthy, full-term infants born at Saint Mary’s Hospital. Samples used were collected from six infants between the ages of 13 and 19 months. Compared to faecal samples from premature infants, these samples represent a richer, more complex bacterial community.

### Sample collection

Samples from premature infants were collected by nursing staff from diapers using a sterile spatula, placed in a sterile DNAase- and RNAase-free Eppendorf tube. Samples from term infants were collected by research nurses from diapers using a sterile scoop, placed in a sterile DNAase- and RNAase-free storage pots. Samples were homogenised with sterile microbiology loops prior to DNA extraction, in light of potential within-sample variation [1].

### Bacterial DNA extraction

Faecal samples (200 mg unless otherwise specified) were processed using one of two methods. Faecal samples from premature infants processed for the long-term freezer storage experiment were processed using “Protocol 1”. In brief, enzymatic digestion of the sample with 10 μl lysozyme (75 mg/ml, Sigma) for 30 min at 37 °C was followed by bead-beating on a FastPrep homogeniser using Lysing Matrix B tubes (MP Biomedicals), with a setting of 6.0 m/s for 45 s (in 3 × 15-s pulses)). Samples were incubated with 2.4 μl RNase A (10 mg/ml) and 6 μl proteinase K (20 mg/ml) for 56 °C for 90 min, followed by a further bead-beating 15-s pulse. DNA was recovered using a phenol-chloroform extraction and purified with the QIAamp DNA mini kit (QIAGEN). All other samples were processed with “Protocol 2”. Here, the FastDNA SPIN Kit for Soil (MP Biomedicals) was used, incorporating bead-beating on a FastPrep homogeniser and performed following the manufacturer’s protocol except that the final elution step was into TRIS (10 mM) low-ethylenediaminetetraacetic acid (EDTA) (0.1 mM) buffer. Negative controls (buffer, no added faecal sample) were processed similarly.

### Polymerase chain reaction amplification and sequencing of variable regions of the bacterial 16S rRNA gene

In this study, we employed two next-generation sequencing platforms—Roche 454 FLX and Illumina Miseq.

For samples analysed using the 454 FLX platform (samples involved in the sample weight experiment), the V3–V5 region of bacterial 16S rRNA genes was amplified from each DNA sample in quadruplicate using a primer pair tagged with individually unique 12-bp error-correcting Golay barcodes [11, 12]. Polymerase chain reaction (PCR) was performed as previously described [10]. Replicate amplicons were pooled and purified, and pyrosequencing runs were carried out on a 454 Life Sciences GS FLX (Roche) following the Roche Amplicon Lib-L protocol. Replicate samples spread over all sequencing runs acted as internal controls.

For samples analysed using the Illumina Miseq platform (all other samples), the V4 region was amplified using the primers 520F “AYTGGGYDTAAAGNG” and 802R “TACNVGGGTATCTAATCC” [13], following a dual barcoding approach [14] using 8-bp Nextera Version 2 barcodes (Illumina). Quadruplicate PCR reactions were performed as follows: reaction mixes consisted of 12.5 μl of Q5 Master Mix (New England BioScience), 1 μl of extracted DNA, 5 μl of each primer pair at 1.5 μM and 1.5 μl of ultrapure water (Cambio). Thermocycler was operated at 95 °C for 2 min, followed by 35 cycles of 95 °C for 20 s, 50 °C for 20 s and 72 °C for 5 min. The reactions were then pooled and purified with sequencing carried out on a Miseq desktop sequencer (Illumina) using a 10 pM library and a 15 % PhiX spike-in, following the standard protocol for Nextera dual-indexing sequencing with V2 kits.

Negative controls were included in all sequencing runs to identify potential contamination.

### Bioinformatics

Four hundred fifty-four shotgun-processed data were denoised using AmpliconNoise [15] as part of the “Quantitative Insights Into Microbial Ecology” (QIIME) [16] package followed by chimera removal with Perseus [15] and demultiplexing. QIIME was also used to join dual-indexed Illumina reads into single reads and demultiplex, with chimera removal performed by USEARCH [17]. Sequences were clustered at 97 % sequence identity using the UCLUST algorithm [17] into operational taxonomic units (OTUs) and aligned by reference to the SILVA rRNA database 119 release [18] (separate processing for each platform). The OTU tables were filtered to remove singletons (sequences present only once in the dataset). Rarefaction was performed, removing heterogeneity of sequencing reads per sample (1622 reads for 454 data, 4356 reads for Illumina). The 454 dataset comprised 294 OTUs in total, and the Illumina dataset comprised 5625 OTUs. Measures of alpha diversity (Shannon-Weaver index, Chao1, total OTUs and phylogenetic diversity) and beta diversity (unweighted and weighted UniFrac distances, Bray-Curtis dissimilarity and Jaccard dissimilarity) were calculated. The rarefied OTU tables were summarised separately to phylum level using the QIIME script summarize_taxa.py.

### Data availability

The datasets supporting the conclusions of this article are available in the European Nucleotide Archive repository, PRJEB6345 (454 data, http://www.ebi.ac.uk/ena/data/view/PRJEB6345) and PRJEB10940 (Illumina data, http://www.ebi.ac.uk/ena/data/view/PRJEB10940).

### Statistics

Alpha and beta diversity measures were analysed using general linear models (GLMs) to determine associations with tested factors. OTUs were analysed using GLMs with a negative binomial distribution. Analysis was performed for all OTUs comprising reads >1 % of total reads from all samples in the involved dataset and OTUs comprising >5 % of any individual sample’s reads. Where significant associations were found, further analysis compared the baseline (the earliest timepoint or the standard weight) and each subsequent category. These analyses were performed using the Wilcoxon signed-rank test for paired data and the Mann-Whitney *U* test for unpaired data (each due to small sample sizes and uneven degrees of variance in the data). The extraction similarity and mail comparison analyses were performed solely using the Mann-Whitney *U* test (rather than GLMs) due to the small numbers of samples involved. Beta diversity measures were compared to standard values derived from replica sequenced samples (see below) using the Mann-Whitney *U* test.

GLMs included sample infant identification number as an additional variable. *P* values are presented prior to multiple hypothesis correction (MHC) where applicable, with data tables indicating whether results would still be significant after a Bonferroni correction.

### Establishing baseline variation

In order to compare the amount of variation arising from experimental conditions, baselines of ‘expected variation’, where a sample is sequenced multiple times, were established for each platform. Samples sequenced were as follows:

- Illumina Miseq: ten replicate samples from infant 14 were sequenced on a single Miseq run.
- Roche 454 FLX: 42 samples were sequenced on two different sequencing runs.
- Beta diversity measures for these baseline samples (henceforth described as “standard” variation between the microbial communities of a single faecal sample that is sequenced twice) are shown in Additional files 1 (Illumina Miseq) and 2 (Roche 454 FLX).

### Experimental summary

To determine the amount of variation that can be expected due to the DNA extraction process, one faecal sample from a term infant (Infant 14) was homogenised and split into ten aliquots. Both sets of five aliquots were frozen at −80 °C, with the first set being extracted after storage for 24 days, and the second set being extracted after storage for 115 days. A separate researcher performed a DNA extraction on each set of aliquots, using extraction kits with a different lot number. The aim of this process was to simulate ‘typical’ laboratory operation, incorporating the differences that would occur when performing multiple DNA extractions (time delays, different staff members and different kit reagents).

To investigate the effects of varying sample weight, fresh faecal samples from four premature babies were each split into four aliquots (25, 50, 100 and 200 mg) prior to freezing at −80 °C for up to 3 weeks before DNA extraction and sequencing.

To investigate the effects of long-term freezer storage, fresh faecal samples from four premature babies were each split into five aliquots, left at room temperature for between 4 and 8 h (simulating the potential collection and processing time period) and then frozen at −80 °C for between 2 months and 2 before DNA extraction and sequencing.

To investigate the effects of room temperature storage, five faecal samples from term infants and four from preterm infants were split into aliquots and stored at room temperature. At regular timepoints, an aliquot of faeces from each infant was moved to −80 °C and the entire set underwent DNA extraction 2 months after the experiment began.

To identify variation that may arise from the mailing of faecal samples, samples from five term babies were split into three aliquots. One aliquot from each baby was mailed to the laboratory from varied locations across London. Two aliquots were left at room temperature, one of which was frozen at −80 °C after 4 h and the other (a mail match) was frozen along with the mailed aliquot when it arrived back at the laboratory.

A summary of the experiments conducted are shown in Table 1.

**Table 1:** Summary of study experiments

Experiment	Subject population	Faecal sample source	DNA extraction protocol	Sequencing region	Sequencing platform
Variation between extractions	Term infants	Infant 14	2	Vv4	Illumina Miseq
Effects of varied weight	Premature infants	Infants 1, 2, 3 and 4	2	V3–V5	Roche 454 GS FLX
Effects of long term freezing	Premature infants	Infants 5, 6, 7 and 8	1	V4	Illumina Miseq
Effects of room temperature storage	Premature and term infants	Infants 5, 6, 7, 8, 9, 10, 11, 12 and 13	2	V4	Illumina Miseq
Effects of mailing samples	Term infants	Infants 9, 10, 11, 12 and 13	2	V4	Illumina Miseq

Experiments performed to determine the effects of different sources of variation. Term infant faecal communities represent more complex microbial mixtures, whilst premature faecal samples harbour more simple communities and are obtained in very limited quantities

## Results

### How much variation can be expected between extractions?

To investigate the validity of comparing samples undergoing separate DNA extractions over the course of a typical laboratory schedule (differing people, dates and kit lot numbers), a single sample was split into ten aliquots and processed in two batches.

**Fig. 1:**
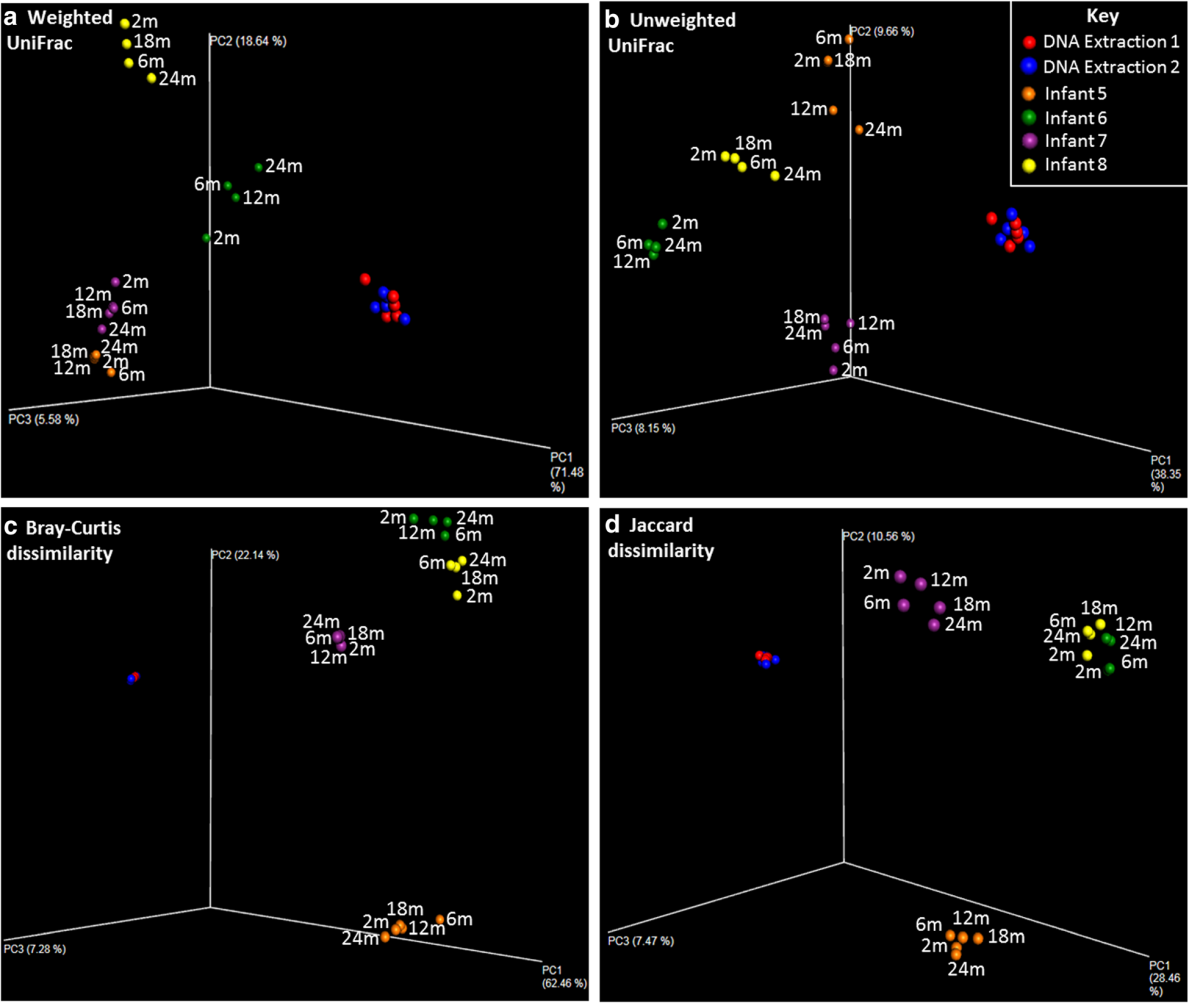
PCoA plots of four beta diversity measures (panels **a**-**d**; **a** - Weighted UniFrac, **b** - Unweighted Unifrac, **c** - Bray-Curtis dissimilarity, **d** - Jaccard dissimilarity), displaying results from two experiments: (i) *red* and *blue points* indicate the samples used for the DNA extraction similarity experiment, where a single faecal sample from a term infant (infant 14) was split into ten aliquots. Samples are grouped according which the DNA extraction they were processed in; each group underwent the same protocol, but was processed by a different researcher, using FastDNA SPIN Kits for soil (MP Biomedicals) with different lot numbers. Samples were frozen at −80 °C prior to extraction and processed after 24 days (DNA extraction 1, *red*) or 115 days (DNA extraction 2, *blue*), simulating typical staggered collection and DNA extraction of samples. (ii) *Orange*, *green*, *purple* and *yellow points* indicate samples included in the long-term storage experiment, where samples from four infants were split into aliquots and stored at −80 °C for short-term (2 months) storage or at −80 °C for ‘long-term’ (6, 12, 18 and 24 months) storage prior to DNA extraction. *Points* are coloured by infant and labelled with the length of storage

**Fig. 2:**
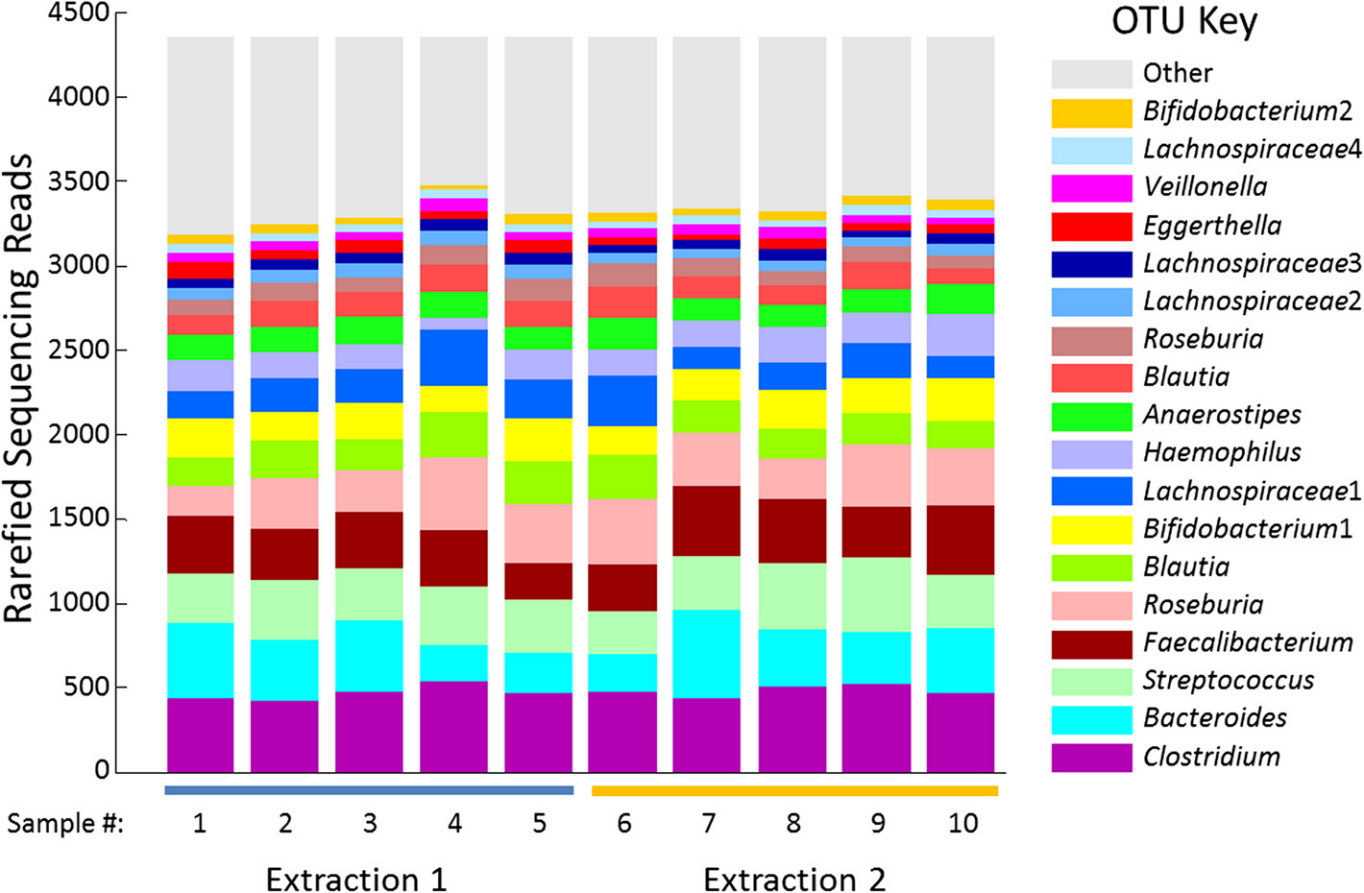
The microbial communities of the samples involved in the DNA extraction similarity experiment, grouped by DNA extraction. Only one OTU, *Lachnospiraceae*2 was found to be differentially abundant between the two groups

No significant differences in any measure of alpha diversity were found between extraction groups. The magnitudes of all measures of beta diversity between samples from different extraction groups were no greater than the magnitudes within extraction groups. Principal coordinates analysis (PCoA) plots of beta diversity measures indicated no segregation of samples by extraction group (see Fig. 1). Comparison of all OTUs identified only a single OTU that differed significantly between the groups (see Fig. 2); *Lachnospiraceae*2 comprised 1.97 % of reads in extraction 1 and 1.45 % in extraction group 2 (*p* = 0.012 prior to multiple hypothesis correction for 18 OTUs tested). No differences in abundance at phyla level were found between groups (see Additional file 3: Figure S1).

### How small an amount of sample is sufficient?

A common problem facing studies that make use of precious samples that are obtainable only in small quantities is the issue of standardisation of sample weight. Such samples are too valuable to be ignored, but their inclusion risks potential weight related bias through undersampling. Alternatively, studies may utilise samples that prove extremely difficult to portion into desired weights; 200–300 mg is a commonly used range of sample weight, although this may be impossible to obtain in some studies. To investigate potential effects, four faecal samples from premature infants were split into a variety of weights prior to DNA extraction and sequencing and the resulting bacterial communities compared.

**Fig. 3:**
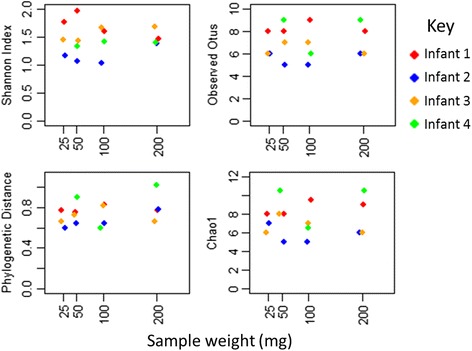
Alpha diversity measures for samples included in the weight variation experiment; four samples (one from each of infants 1–4) were split into aliquots of varying weights prior to DNA extraction and sequencing (which was performed on a 454 FLX). *Points* are coloured by infant. No significant associations between reduced weight and changes in alpha diversity were found

**Fig. 4:**
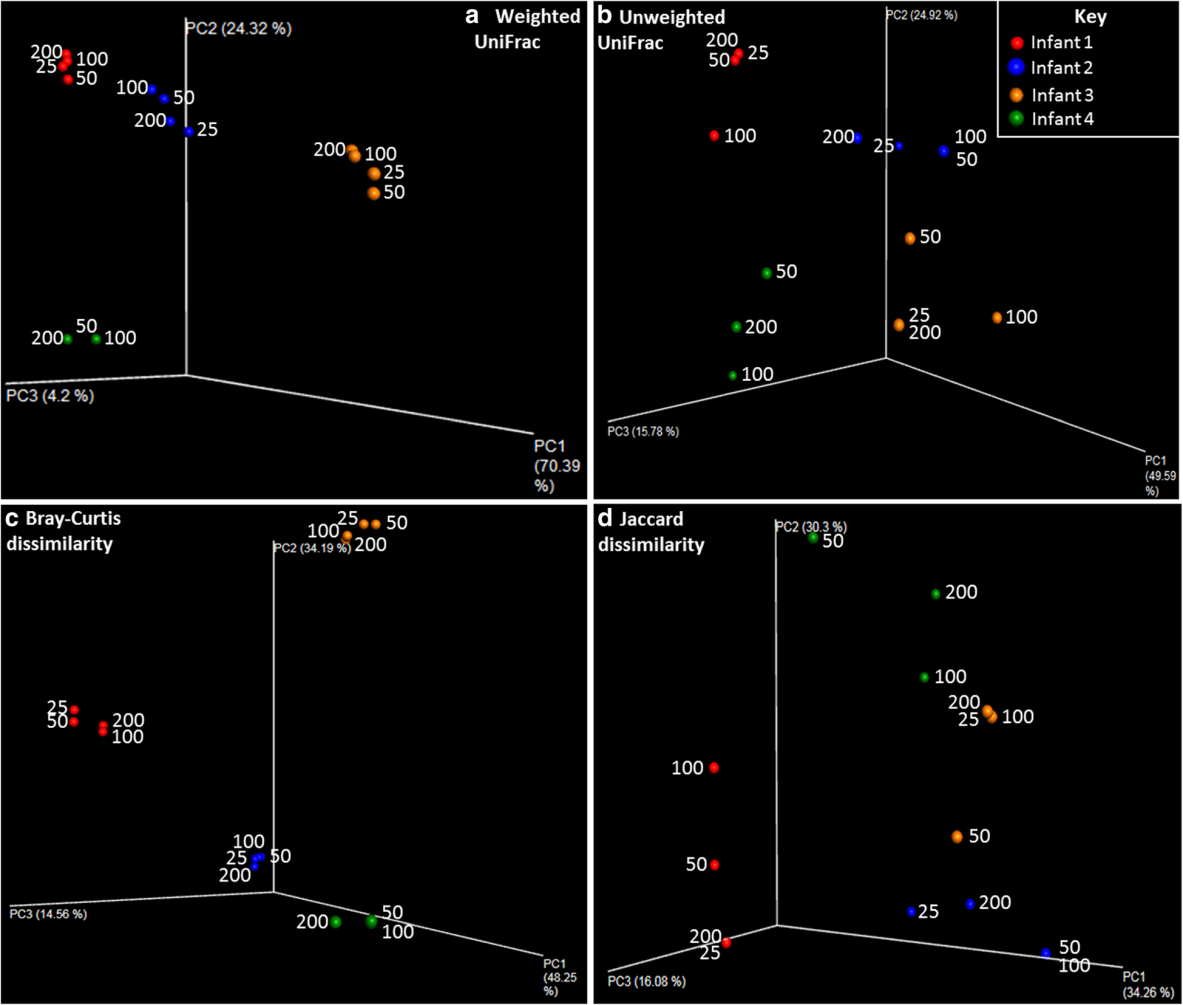
PCoA plots of four beta diversity measures (panels **a**-**d**; **a** - Weighted UniFrac, **b** - Unweighted Unifrac, **c** - Bray-Curtis dissimilarity, **d** - Jaccard dissimilarity) for samples included in the weight variation experiment. Each *point* is coloured by infant and labelled with the weight of the sample used

GLMs found no significant associations between reduced sample size and shifts in any measure of alpha diversity (see Fig. 3). GLMS also found no association between reduced weight and increased beta diversity measures, although comparison to the standard values indicated that weighted UniFrac distances were significantly higher than expected (*p* = 0.011). Standard values of beta diversity were compared to the magnitudes between the 200 mg and each set of sample weights in turn. Higher than expected values were found for only one comparison; weighted UniFrac distances were significantly higher than expected when sample size was reduced to 50 mg (*p* = 0.024 prior to MHC for 16 tests performed), although there was no significant difference when comparing the 200 mg samples to the 25 mg set (comparisons shown in Additional file 4: Figure S2). PCoA plots of weighted UniFrac distances and Bray-Curtis dissimilarity display tight clustering of the samples despite higher than expected variation, whilst unweighted UniFrac and Jaccard dissimilarity based clustering is looser due to the loss and gain of low abundance OTUs (comprising <5 % of sample reads) (see Fig. 4), but still within the expected range. The derived microbial communities are shown in Additional file 5: Figure S3.

### What are the effects of long term freezing?

The current ‘gold standard’ for long-term storage of samples is assumed to be freezing at −80 °C. However, studies have not investigated the potential long term effects on the microbial community. Given that studies may take years to collect samples, and ideally will prove to be a reliable resource for years to come, it is essential to quantify any variation that may occur. We investigated the effects of storage at −80 °C for up to 2 years on the derived microbial community.

**Fig. 5:**
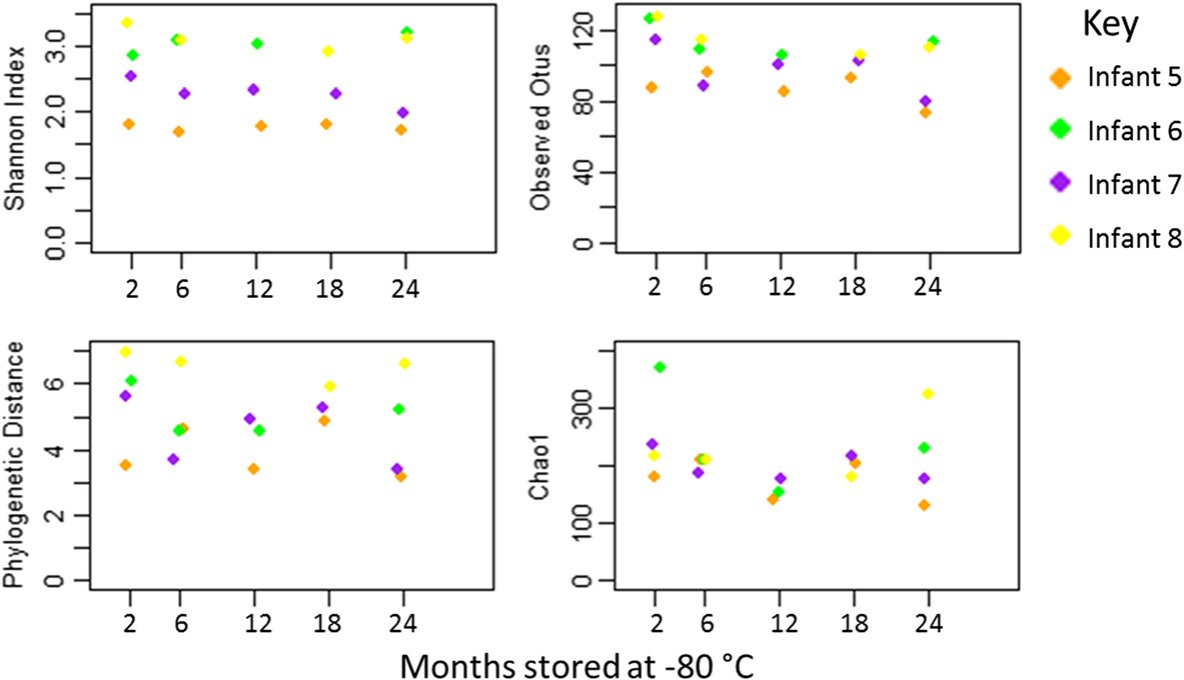
Alpha diversity measures samples included in the long-term storage experiment. *Points* are coloured by infant. The total number of observed OTUs was found to decrease significantly with increased duration of storage

Of the four measures of alpha diversity, GLMs found an association between months of storage and only one measure, total observed OTUs, which decreased over time (*p* = 0.018 prior to multiple hypothesis correction for four tests, GLM coefficient of −0.660 (−0.189, −1.140), a loss of 7.92 (2.16, 13.69) observed OTUs per year of storage) (see Fig. 5). Comparisons of the alpha diversity measures observed at two months with later timepoints indicated no significant differences.

GLMS found no association between longer term storage and increases in beta diversity measures. PCoA plots generally showed clustering of later timepoints around the 2-month sample consistent with random variation (see Fig. 1). The weighted UniFrac distances of 24-month samples from three of four babies were usually high however, indicating substantial changes in the abundance of some bacterial genera. GLMs were used to test for significant shifts in specific OTUs over time. Of the 24 OTUs tested, 8 had significant changes in relative abundance (see Table 2).

**Table 2:** OTUs with differential abundances over long term storage at −80 °C

	Mean reads (% of group total) at:						Significant after a MHC?
OTU	2 months	24 months	Exponentiated coefficient	2.5 % CI	97.5 % CI	*P* value	
*Lactobacillus*1	3.306	5.596	1.021	1.012	1.029	>0.001	Yes
*Lactobacillus*2	0.109	0.258	1.035	1.014	1.056	0.001	Yes
Bacilli	0.585	0.735	1.013	1.001	1.026	0.028	No
*Enterobacteriaceae*	0.683	0.218	0.948	0.934	0.962	>0.001	Yes
Gammaproteobacteria1	0.620	0.304	0.970	0.955	0.986	>0.001	Yes
*Enterococcus*2	0.207	0.396	1.031	1.014	1.048	>0.001	Yes
*Staphylococcus*1	0.247	0.189	0.975	0.956	0.994	0.009	No
*Staphylococcus*2	0.339	0.143	0.967	0.944	0.990	0.005	No

The OTUs described shift significantly over the course of long term storage at −80 °C. Mean sequencing reads in the samples stored for 2 months and those stored for 24 months are shown. The exponentiated coefficients were provided by the GLMs and approximate the rate of change in relative abundance for each OTU per month of storage; 2.5 and 95 % CI indicate the 95 % confidence interval around the exponentiated coefficient. *P* values indicate the significance of the coefficient (prior to MHC for 24 tested OTUs).The final column indicates whether the result would still be significant after a Bonferroni correction

**Fig. 6:**
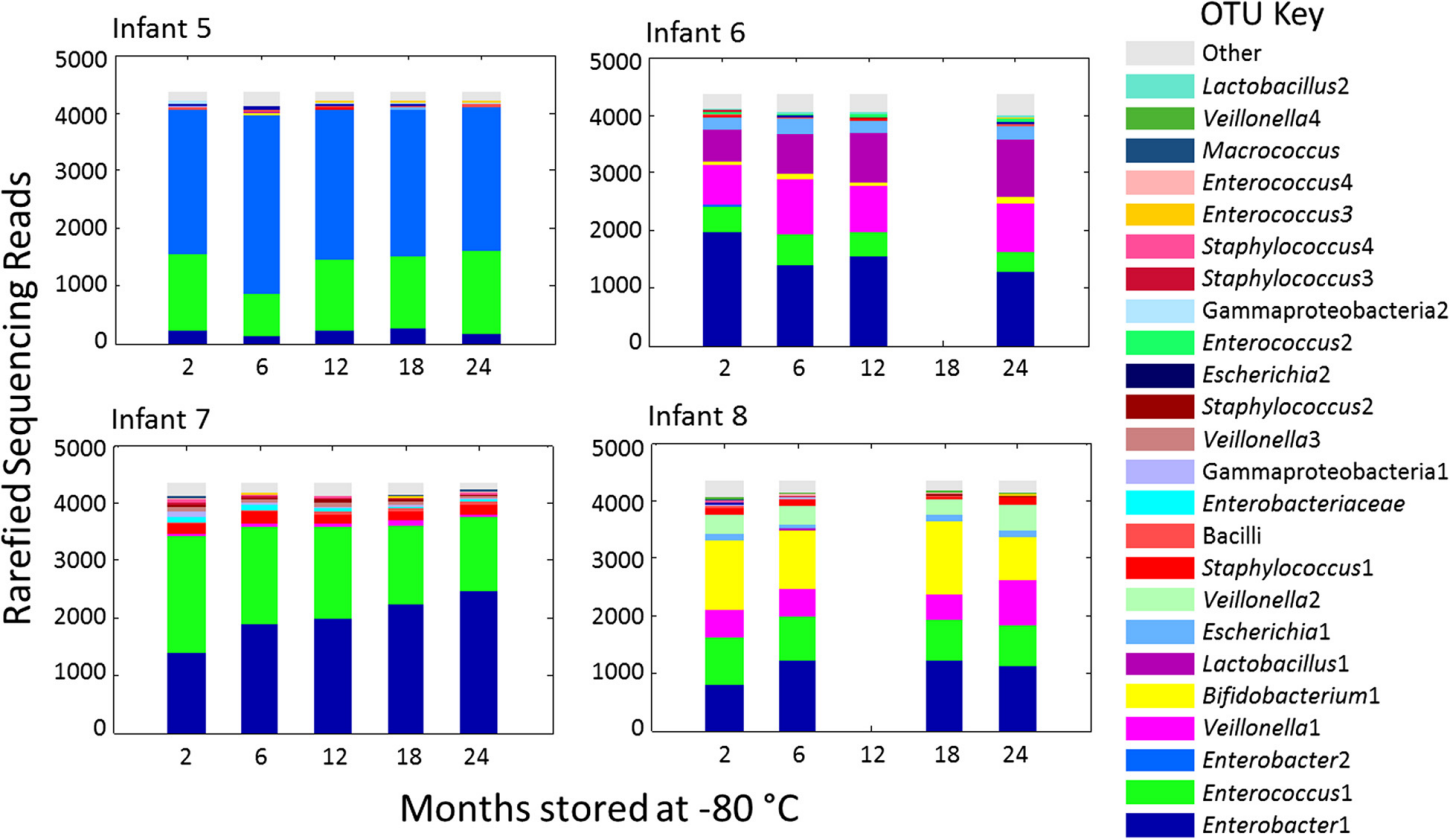
The microbial communities of the samples included in the long-term storage experiment. OTUs with relative abundance that was significantly associated with storage duration are described in Table 2

Whilst OTUs representing some genera shift in concert (both *Lactobacillus* OTUs increasing over time, both *Staphylococcus* OTUs decreasing), other changes were not shared amongst similar OTUs (four other *Enterobacteriaceae* and one other Gammaproteobacteria OTUs did not change significantly and only one of four *Enterococcus* OTUs decreased over time). The OTUs tested are shown in Fig. 6. No significant differences were found for any OTUs when directly comparing the 2-month timepoint to other timepoints. Analysis of bacterial phyla indicated no significant shifts due to long-term storage (microbial communities summarised to phyla are shown in Additional file 6: Figure S4).

### What is the effect of room temperature storage on microbial communities?

Concerns have been raised concerning the stability of DNA when samples are kept at room temperature and resulting effects on microbial communities. Whilst studies have looked at specific timepoints, the time prior to storage of samples at −80 °C will be likely to vary greatly in studies involving hundreds of samples. We investigated the effects of room temperature storage on both simple and relatively complex communities (pre-term and term faecal samples, respectively) over a detailed timecourse. The two sample sets were analysed and independently.

**Fig. 7:**
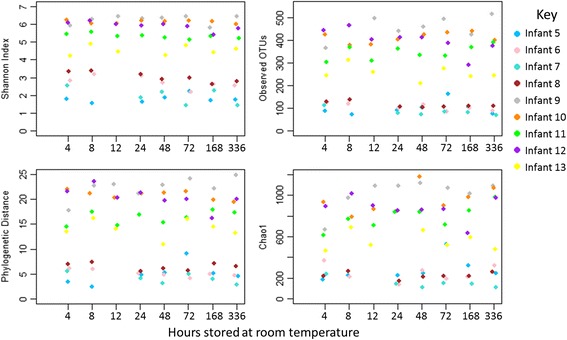
Alpha diversity measures for the room temperature storage experiment. Samples from four premature infants (infants 5–8) and five term infants (infants 9–13) were split into aliquots and stored at room temperature for between 4 h and 2 weeks prior to transfer to −80 °C storage and DNA extraction. *Points* are coloured by infant. A significant decrease in the Shannon index over time was observed for the premature infant samples

Of the alpha diversity measures, only the Shannon index was found shift significantly over time, decreasing in the low complexity samples (*p* = 0.029, GLM coefficient of −0.0012 (−0.0022, −0.0002) equating to a 0.0288 (0.0530, 0.0047) reduction in the Shannon index per day of room temperature storage). A non-significant decrease was observed in the complex dataset. Alpha diversity timecourses are shown in Fig. 7.

**Fig. 8:**
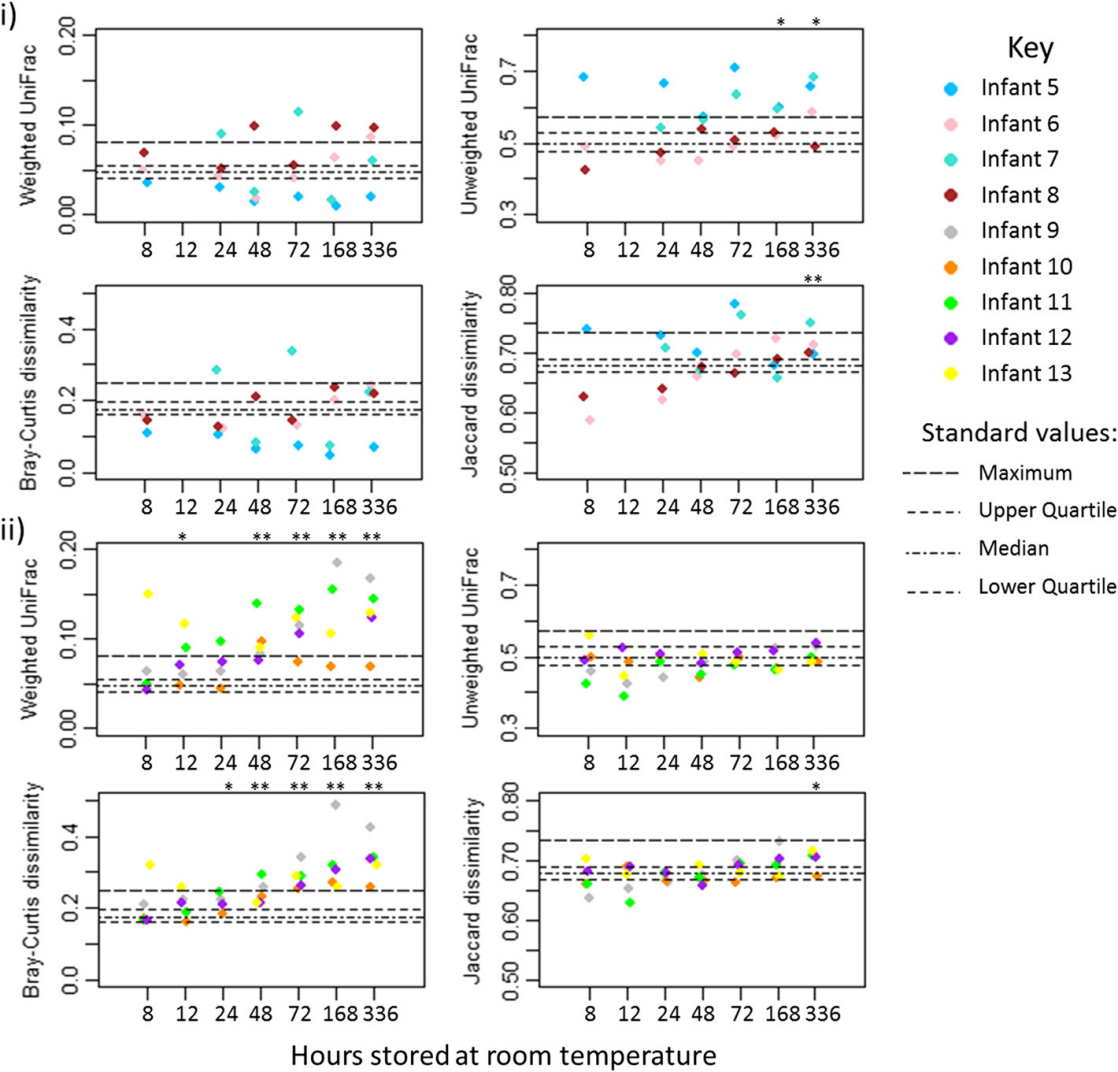
Beta diversity measures for the room temperature storage experiment. Magnitudes of the different measures (indicated on the *y* axes) are quantified between the aliquots stored for 4 hours at room temperature and aliquots stored for the time indicated on the *x* axis. *Stars* above timepoints indicate any significant difference (determined by the Mann-Whitney *U* test) when comparing the magnitudes at that timepoint to the standard values; *p value of <0.05, **p value of <0.01. Simple and more complex communities were tested separately with **i** showing timecourses for simple community samples and **ii** showing time courses for more complex community samples. *Points* are coloured by infant and metrics summarising the spread of the standard values are indicated by the *dashed lines*

Increased time from the 4-h timepoint was associated with significant increases in unweighted UniFrac distances for the simple community samples (*p* = 0.038), whilst the more complex samples displayed a significant increase over time for all beta diversity measures (weighted UniFrac, *p* < 0.001; unweighted UniFrac, *p* = 0.049; Bray-Curtis dissimilarity, *p* < 0.001; Jaccard dissimilarity, *p* < 0.001). Comparison of individual timepoints to standard beta diversity values indicated that the simple communities had significantly increased unweighted UniFrac distances by 1 week of storage, and the more complex communities had significantly increased weighted UniFrac distances at 12 and 48 h onwards. Bray-Curtis dissimilarity was significantly higher for the more complex communities from 24 h of storage. Jaccard dissimilarity was also significantly higher than the standard values for both datasets by 2 weeks (see Fig. 8). PCoA plots for beta diversity measures are shown in Additional file 7: Figures S5, S6, S7 and S8.

Analysis of OTUs indicated a significant association (prior to MHC) between relative abundance and duration of room temperature storage for 4 of 9 tested OTUs in the simple dataset (see Table 3) and 12 of the 31 tested OTUs in the complex dataset (see Table 4). Common trends between the datasets were increased relative abundances of *Bifidobacterium* and decreased *Veillonella*.

**Table 3:** OTUs in the simple community dataset that displayed differential abundances over the course of two weeks of storage at room temperature

	Mean reads (% of dataset total) at:						Significant after a MHC?
OTU	4 h	2 weeks	Exponentiated coefficient	2.5 % CI	97.5 % CI	*P* value	
*Enterococcus*	26.61	34.00	1.0008	1.0001	1.0015	0.034	No
*Bifidobacterium*	7.15	10.78	1.0028	1.0010	1.0046	0.002	Yes
*Veillonella*1	7.06	1.12	0.9936	0.9903	0.9968	<0.001	Yes
*Veillonella*2	2.03	0.09	0.9892	0.9868	0.9916	<0.001	Yes

The OTUs shown shift significantly in relative abundance in the simple faecal community dataset over 2 weeks of storage at room temperature. Mean sequencing reads in the samples stored for 4 h and those stored for 2 weeks are shown. The exponentiated coefficients were provided by the GLMs and approximate the rate of change of relative abundance for each OTU per hour of storage; 2.5 and 95 % CI indicate the 95 % confidence interval around the exponentiated coefficient. *P* values indicate the significance of the coefficient (prior to MHC for nine tested OTUs). The final column indicates whether the result would still be significant after a Bonferroni correction

**Table 4:** OTUs in the more complex community dataset that displayed differential abundances over the course of 2 weeks of storage at room temperature

	Mean reads (% of dataset total) at:						Significant after a MHC?
OTU	4 h	2 weeks	Exponentiated coefficient	2.5 % CI	97.5 % CI	*P* value	
*Bifidobacterium*1	6.82	7.25	1.0005	1.0001	1.0010	0.022	No
*Enterobacter*	0.89	6.44	1.0031	1.0009	1.0053	0.006	No
*Bifidobacterium*3	2.84	3.96	1.0012	1.0005	1.0018	<0.001	Yes
*Bacteroides*2	1.32	2.38	1.0013	1.0004	1.0022	0.005	No
*Prevotella*	2.20	0.14	0.9910	0.9876	0.9945	<0.001	Yes
*Subdoligranulum*	1.49	0.80	0.9978	0.9964	0.9992	0.002	No
*Peptostreptococcaceae*1	1.82	0.83	0.9974	0.9962	0.9986	<0.001	Yes
*Peptostreptococcaceae*2	0.77	1.93	1.0022	1.0012	1.0032	<0.001	Yes
*Anaerostipes*1	1.66	0.79	0.9987	0.9978	0.9996	0.004	No
*Veillonella*	1.08	0.55	0.9951	0.9936	0.9966	<0.001	Yes
*Ruminococcus*	1.12	1.40	1.0008	1.0002	1.0013	0.004	No
*Pseudobutyrivibrio*	1.38	0.18	0.9957	0.9938	0.9977	<0.001	Yes

The OTUs shown shift significantly in abundance in the more complex faecal community dataset over two weeks of storage at room temperature. Column descriptors as noted for Table 3

**Fig. 9:**
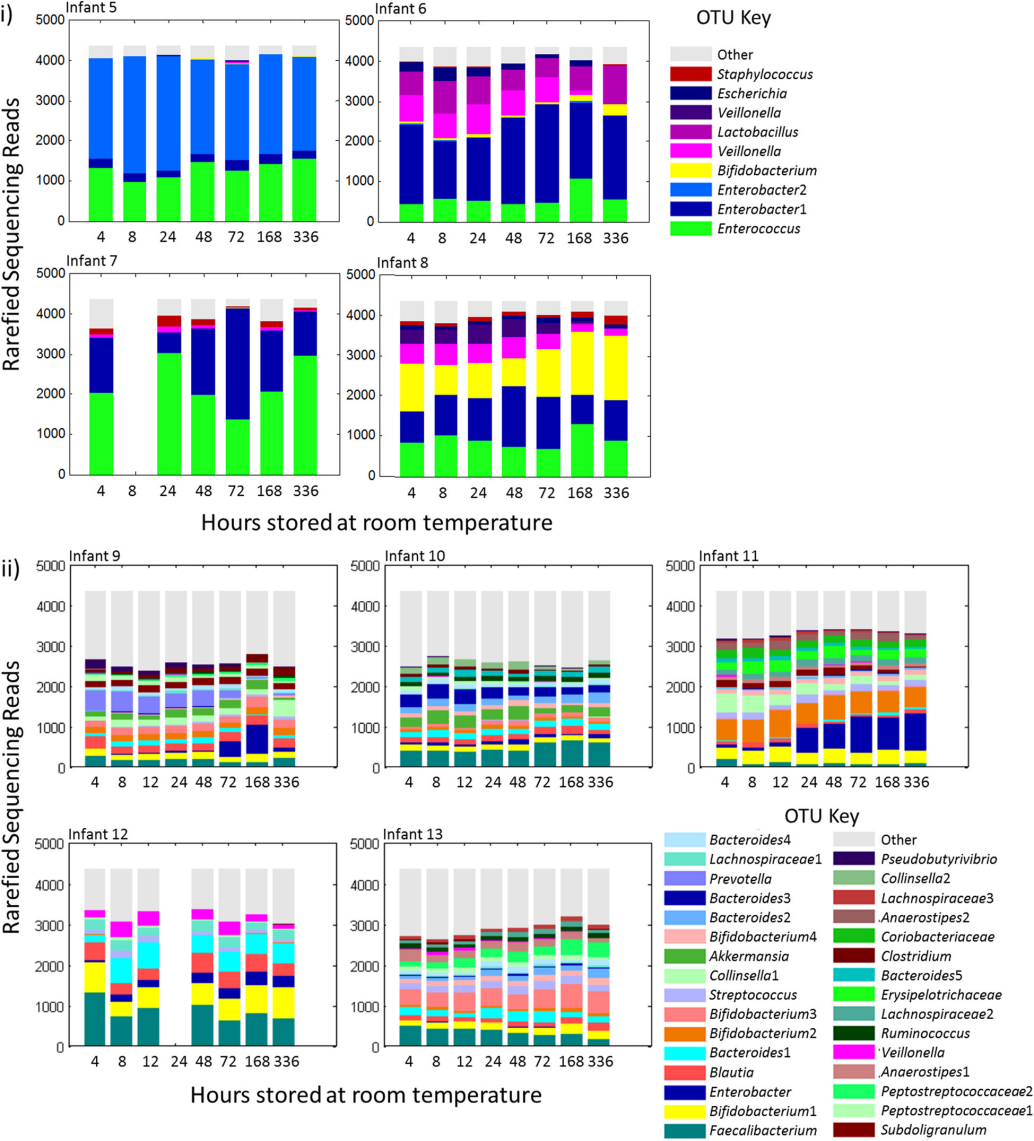
The microbial communities of the samples included in the room temperature storage experiment, derived from OTUs. Samples are divided into the two analysed groups: **i** faecal samples from premature babies with low complexity communities. **ii** Faecal samples from term infants with more complex communities. OTUs with relative abundance that was significantly associated with storage duration are described in Tables 3 and 4

Timecourses of the OTUs analysed are shown in Fig. 9. At the level of phyla, longer storage at room temperature was associated with a significant decrease in Firmicutes (*p* = 0.004) and an increase in Proteobacteria (*p* = 0.018) in the complex faecal communities, in addition to a significant increase in Actinobacteria in simple and complex communities (*p* values of 0.013 and 0.033, respectively). Only the shift in Firmicutes was significant after MHC (with an exponentiated coefficient of 0.9997 (0.9994, 0.999), 62.9 % of mean dataset reads at 4 h, 52.9 % at 2 weeks), and no similar shift was observed in the simple community (for phyla level microbial community timecourses, see Additional file 8: Figure S9).

### Does the mailing of faecal samples lead to microbial community divergence?

Environmental or population-based studies often require the collection of samples over a wide-geographic area. In such scenarios, sample transfer via the mail system can prove to be and extremely useful method for returning samples to the laboratory. Whilst we have shown that if the microbial community is relatively stable at room temperature for up to 2 days, the mail system itself may be a source of considerable environmental variation—our own university mail room has been known to reach 30 °C (86 °F) on an otherwise cool September day.

Mailed aliquots of samples were compared to two paired aliquots. One was stored at room temperature for 4 h prior to −80 °C storage (baseline samples). The second (a mail match) was stored at room temperature until the paired mail aliquot arrived at the laboratory. No significant differences in alpha diversity were found between the mail and mail match sample groups. Measures of beta diversity between baseline samples and each group were not significantly different (see Additional file 7: Figures S5, S6, S7 and S8 for PCoA plots). No differentially abundant OTUs were found between the mail and mail match groups (see Additional file 9: Figure S10), and no differences were found in phyla abundance (see Additional file 10: Figure S11).

## Discussion and conclusion

A number of studies have looked at sample compositional stability associated with different conditions of storage. Carroll et al. [2] have provided the current benchmark for long-term −80 °C storage, showing no significant changes in the faecal microbial community after 6 months of storage, with Bai et al. [3] reporting similar findings for the vaginal microbiota after a shorter 4 weeks of storage. For some studies, however, samples may need to be accrued over far longer periods, and the impact of longer term storage has not until now been systematically appraised. Here, we report that 2 years at −80 °C leads to few significant changes in the microbial community of infant faecal samples, with a small reduction in the observed number OTUs and shifts in the abundance of some specific OTUs, although these were often not shared amongst similar lineages. The increases in abundance of the *Lactobacillus* and bacilli OTUs are a notable exception to this trend, and it may be important to consider the effects of long-term storage of samples where studies focus on these organisms. We also note that the results presented may not be representative of more complex mixtures and that the effects of long-term storage have only been described for the specific organisms featuring in our dataset.

Carroll et al. [2] and Cardona et al. [4] have investigated sample stability at room temperature. Both groups demonstrated compositional stability of faecal samples over a 24-h period. A lack of significant change in the relative proportions of different bacterial phyla has been reported over a 3-day period of storage at room temperature by Dominianni et al. [7]. Voigt et al. [1] and Flores et al. [6] have demonstrated stability out to 7 days when samples are preserved in the proprietary Stabilization Solution “RNAlater”. These findings have appeared to represent the limit. Cardona et al. [4] have shown that significant changes in the community are seen in samples analysed after 2 weeks at room temperature. Here, we have investigated the compositional stability of infant faecal samples held at room temperature for periods out to 2 weeks and confirm the findings of Carrol et al., Cardona et al. and Dominianni et al. [2, 4, 7] that by 1 week, significant skewing of the observed population has occurred. In our more complex community samples, this primarily affects the abundance of some elements of the bacterial community, with our analysis indicating significant shifts in the weighted measures of beta diversity by 48 h. The most extreme shifts appeared to be correlated with increasing relative abundance of *Enterobacteriaceae* (specifically an *Enterobacter* OTU). The less complex community samples were more strongly affected by loss and gain of low abundance OTUs, leading to significant increases in unweighted measures. Considerable differences in both bacterial OTUs and phyla were evident by 1 and 2 weeks of room temperature storage, and although no particular trends unified which groups are likely to be affected, increased proportional abundance of *Bifidobacterium* and decreased *Veillonella* were seen in both datasets.

We conclude accordingly that for preservation of bacterial community structure, faecal samples should be frozen within 2 days of collection. In the UK, this happily allows time for samples to be delivered to the laboratory by mail without loss of quality, provided that they are dispatched on the day of collection by first class mail and care is taken to avoid weekends. In settings where the mail service is unreliable, alternative means of sample delivery should be considered to ensure that quality is maintained.

By carrying out replicate DNA extractions (different researchers, different lots of reagents), we have concluded that this part of the overall protocol contributes negligibly to variation.

Reduction in faecal sample weight, even down to only an eighth of the generally applied ‘standard’ of 200 mg, also scarcely affected alpha diversity and the majority of beta diversity measures, noting that this related to our study of infant samples of inherently low diversity. The strongest effects were seen when considering weighted beta diversity measures, with OTU abundances suffering increased variation as lower amounts of faeces are sampled. However, despite variation being higher than expected, the actual magnitude of these changes appears to be relatively small, with samples still retaining close similarity. We consider that it would be prudent to establish on individual study basis a minimum sample size for DNA extraction to yield consistent results.

## Abbreviations

EDTA, ethylenediaminetetraacetic acid; GLM, general linear models; MHC, multiple hypothesis correction; OTU, operational taxonomic unit; PCoA, principal coordinates analysis; PCR, polymerase chain reaction; QIIME, quantitative insights into microbial ecology; rRNA, ribosomal ribonucleic acid; TRIS, tris(hydroxymethyl)aminomethane

## Additional files

Additional file 1: Illumina Miseq expected variation. (XLS 15 kb) Additional file 2: Roche 454 FLX expected variation. (XLS 10 kb) Additional file 3: Figure S1. The microbial communities of the samples used in the DNA extraction similarity experiment, summarised to phyla. (DOCX 70 kb) Additional file 4: Figure S2. Beta diversity measures for the weight variation experiment. Magnitudes of the different measures (indicated on the *y* axes) are quantified between the 200-mg sample and the weight indicated on the *x* axis. Experiment samples (red, blue, orange and green) are compared to standard beta diversity magnitudes (derived from 42 samples sequenced twice, purple). Stars above timepoints indicate any significant difference (determined by the Mann-Whitney U test) when comparing the magnitudes at that timepoint to the standard values; * indicates a *p* value of <0.05. (DOCX 151 kb) Additional file 5: Figure S3. The microbial communities of the samples used in the weight variation storage experiment. (DOCX 108 kb) Additional file 6: Figure S4. The microbial communities of the samples used in the long term storage experiment, summarised to phyla. (DOCX 95 kb) Additional file 7: Figures S5-S8.
**Figure S5.** - PCoA plot using the weighted UniFrac metric. Sample sets for two experiments are shown samples from four premature infants (infants 5–8) and five term infants (infants 9–13) are shown, encompassing two experiments: (i) for the room temperature storage experiment, samples were split into aliquots and stored at room temperature for between 4 h and 2 weeks prior to transfer to −80 °C storage and DNA extraction. Samples are coloured by infant and labelled with the number of hours of storage. (ii) For the mail experiment, two additional aliquots were taken for the five term babies, one of which was mailed to the laboratory (M) and the other stored at room temperature (MM). Upon arrival of the mailed sample, both were frozen at −80 °C storage prior to DNA extraction. **Figure S6.**—PCoA plot using the unweighted UniFrac metric. Samples labelling as Additional file 7 Figure S5. **Figure S7.** —PCoA plot using the Bray-Curtis dissimilarity metric. Samples labelling as Additional file 7: Figure S5. **Figure S8.** —PCoA plot using the Jaccard dissimilarity metric. Samples labelling as Additional file 7: Figure S5. (DOCX 1219 kb) Additional file 8: Figure S9. The microbial communities of the samples used in the room temperature storage experiment, summarised to phyla. From top left to bottom right, infants 5 to 13, with infants 5–8 being premature (simple faecal communities) and 9–13 being term (more complex faecal communities). (DOCX 154 kb) Additional file 9: Figure S10. The microbial communities of the samples used in the mail experiment. (DOCX 97 kb) Additional file 10: Figure S11. The microbial communities of the samples used in the mail experiment, summarised to phyla. (DOCX 83 kb)
