# Supplementary material for: Latitude in sample handling and storage for infant faecal microbiota studies: the elephant in the room?
Source: Microbiome. 2016 Jul 30;4:40. doi: 10.1186/s40168-016-0186-x (PMC4967342; doi:10.1186/s40168-016-0186-x)
Supplement: Additional file 4: Figure S2. — Beta diversity measures for the weight variation experiment. Magnitudes of the different measures (indicated on the y axes) are quantified between the 200-mg sample and the weight indicated on the x axis. Experiment samples (red, blue, orange and green) are compared to standard beta diversity magnitudes (derived from 42 samples sequenced twice, purple). Stars above timepoints indicate any significant difference (determined by the Mann-Whitney U test) when comparing the magnitudes at that timepoint to the standard values; * indicates a p value of <0.05. (DOCX 151 kb) [file 40168_2016_186_MOESM4_ESM.docx]

**Additional file 4: Figure S2**


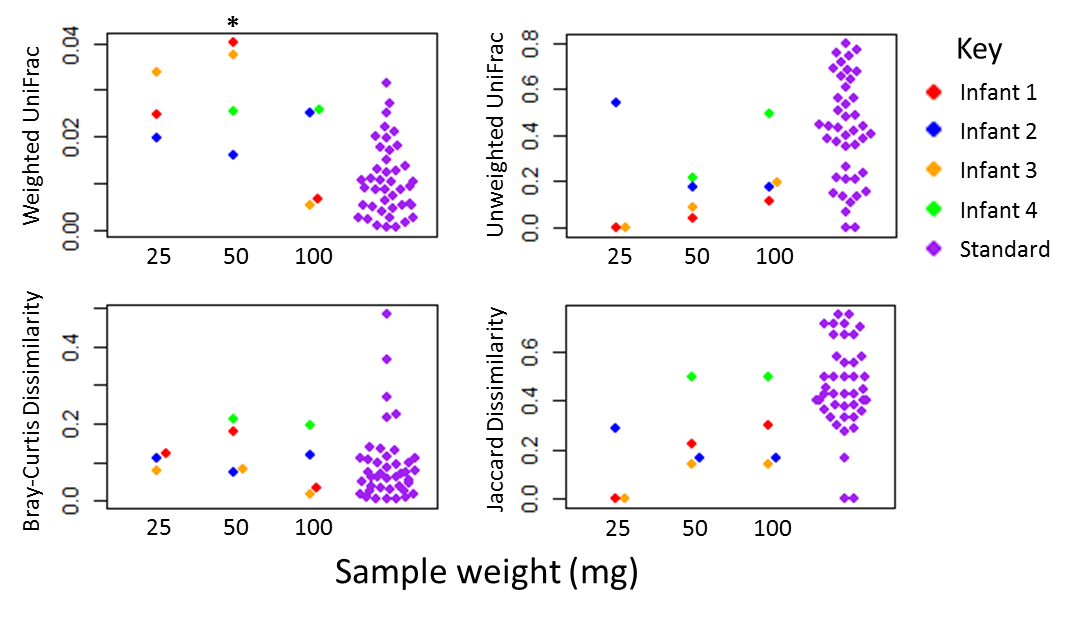


*Additional file 4: Figure S2 - Beta diversity measures for the weight variation experiment. Magnitudes of the different measures (indicated on the y axes) are quantified between the 200 mg sample and the weight indicated on the x axis. Experiment samples (red, blue, orange and green) are compared to standard beta diversity magnitudes (derived from 42 samples sequenced twice, purple). Stars above timepoints indicate any significant difference (determined by the Mann-Whitney U test) when comparing the magnitudes at that timepoint to the standard values; * indicates a p value of <0.05.*
