# Supplementary material for: Latitude in sample handling and storage for infant faecal microbiota studies: the elephant in the room?
Source: Microbiome. 2016 Jul 30;4:40. doi: 10.1186/s40168-016-0186-x (PMC4967342; doi:10.1186/s40168-016-0186-x)
Supplement: Additional file 5: Figure S3. — The microbial communities of the samples used in the weight variation storage experiment. (DOCX 108 kb) [file 40168_2016_186_MOESM5_ESM.docx]

**Additional file 5: Figure S3**
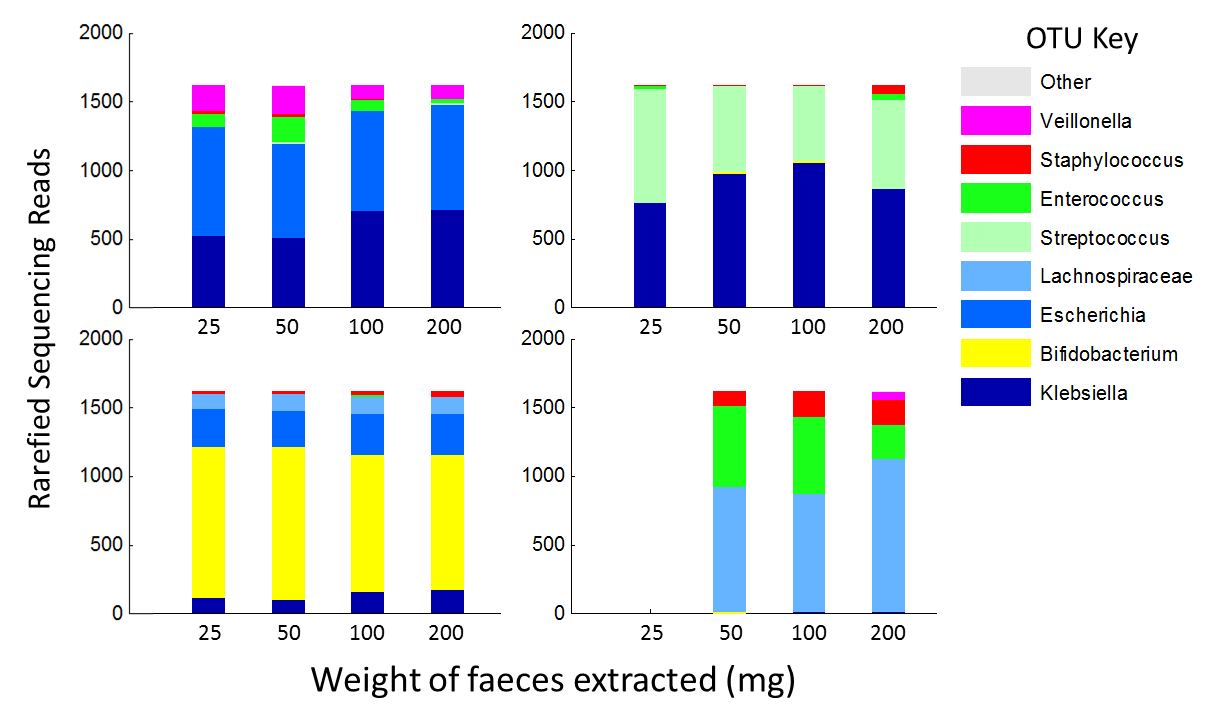


*Figure S3 - The microbial communities of the samples used in the weight variation storage experiment.*
