# Supplementary material for: Latitude in sample handling and storage for infant faecal microbiota studies: the elephant in the room?
Source: Microbiome. 2016 Jul 30;4:40. doi: 10.1186/s40168-016-0186-x (PMC4967342; doi:10.1186/s40168-016-0186-x)
Supplement: Additional file 7: Figures S5-S8. — Figure S5. - PCoA plot using the weighted UniFrac metric. Sample sets for two experiments are shown samples from four premature infants (infants 5–8) and five term infants (infants 9–13) are shown, encompassing two experiments: (i) for the room temperature storage experiment, samples were split into aliquots and stored at room temperature for between 4 h and 2 weeks prior to transfer to −80 °C storage and DNA extraction. Samples are coloured by infant and labelled with the number of hours of storage. (ii) For the mail experiment, two additional aliquots were taken for the five term babies, one of which was mailed to the laboratory (M) and the other stored at room temperature (MM). Upon arrival of the mailed sample, both were frozen at −80 °C storage prior to DNA extraction. Figure S6.—PCoA plot using the unweighted UniFrac metric. Samples labelling as Additional file 7 Figure S5. Figure S7. —PCoA plot using the Bray-Curtis dissimilarity metric. Samples labelling as Additional file 7: Figure S5. Figure S8. —PCoA plot using the Jaccard dissimilarity metric. Samples labelling as Additional file 7: Figure S5. (DOCX 1219 kb) [file 40168_2016_186_MOESM7_ESM.docx]

**Additional file 7: Figure S5**


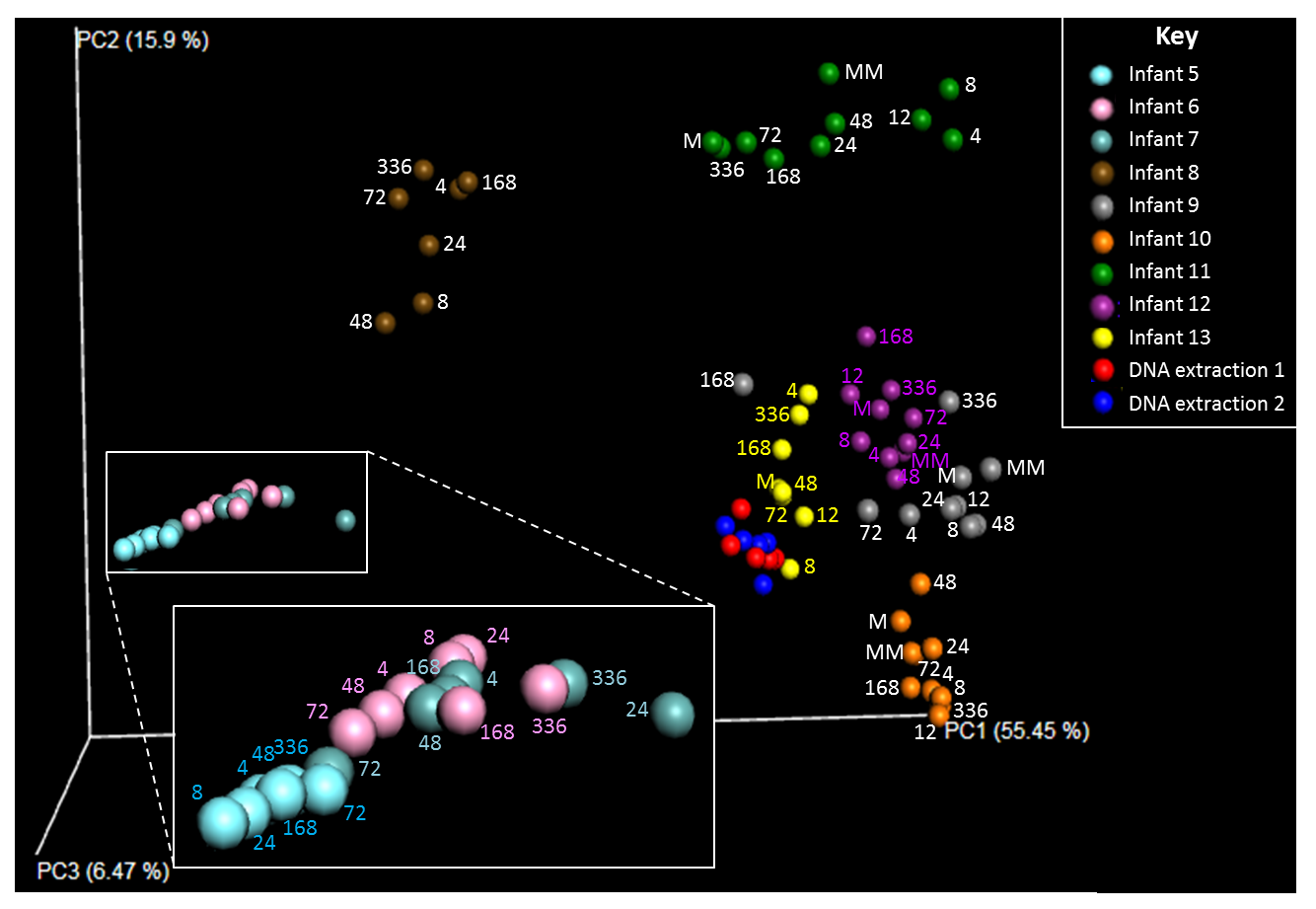


*Additional file 7: Figure S5 – PCoA plot using the weighted UniFrac metric. Sample sets for two experiments are shown Samples from four premature infants (infants 5 – 8) and five term infants (infants 9 – 13) are shown, encompassing two experiments; i) For the room temperature storage experiment, samples were split into aliquots and stored at room temperature for between four hours and two weeks prior to transfer to -80°C storage and DNA extraction. Samples are coloured by infant and labelled with the number of hours of storage. ii) For the mail experiment, two additional aliquots were taken for the five term babies, one of which was mailed to the laboratory (M) and the other stored at room temperature (MM). Upon arrival of the mailed sample, both were frozen at -80°C storage prior to DNA extraction.*

**Figure S6**


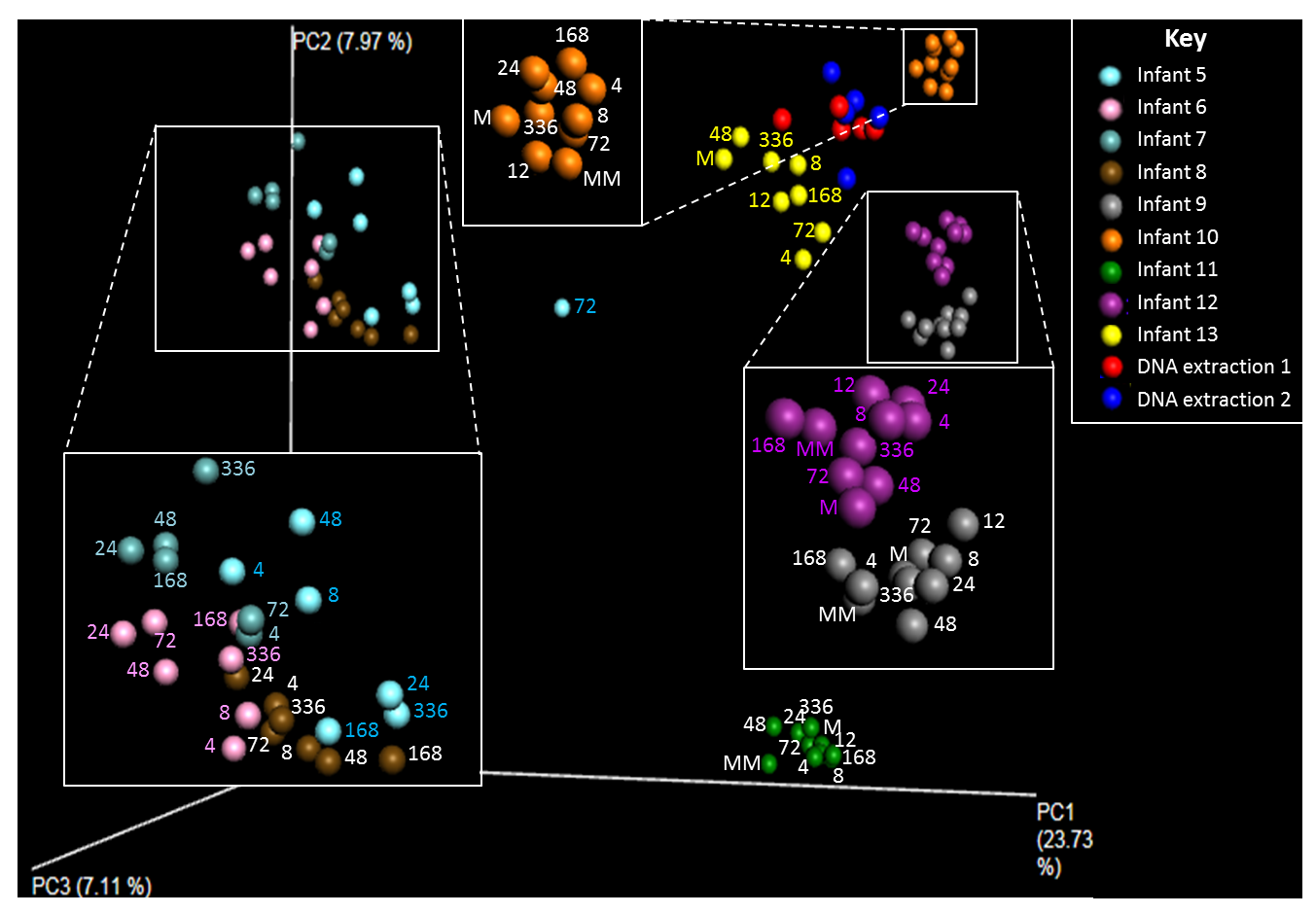


*Figure S6 – PCoA plot using the unweighted UniFrac metric. Samples labelling as Supplementary Figure 5.*

**Figure S7**

*
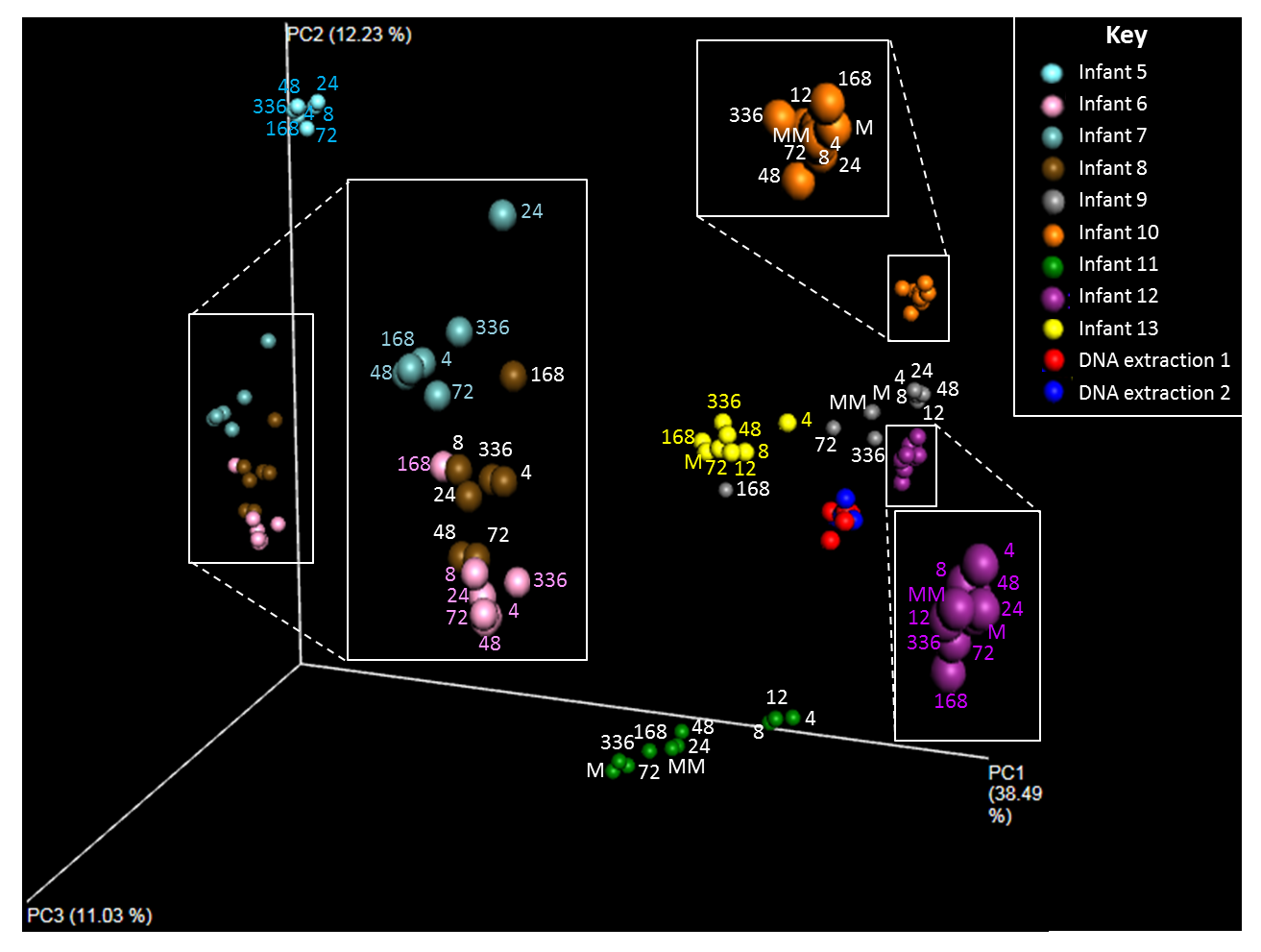
*

*Figure S7 – PCoA plot using the Bray-Curtis dissimilarity metric. Samples labelling as Supplementary Figure 5.*

**Figure S8**

*
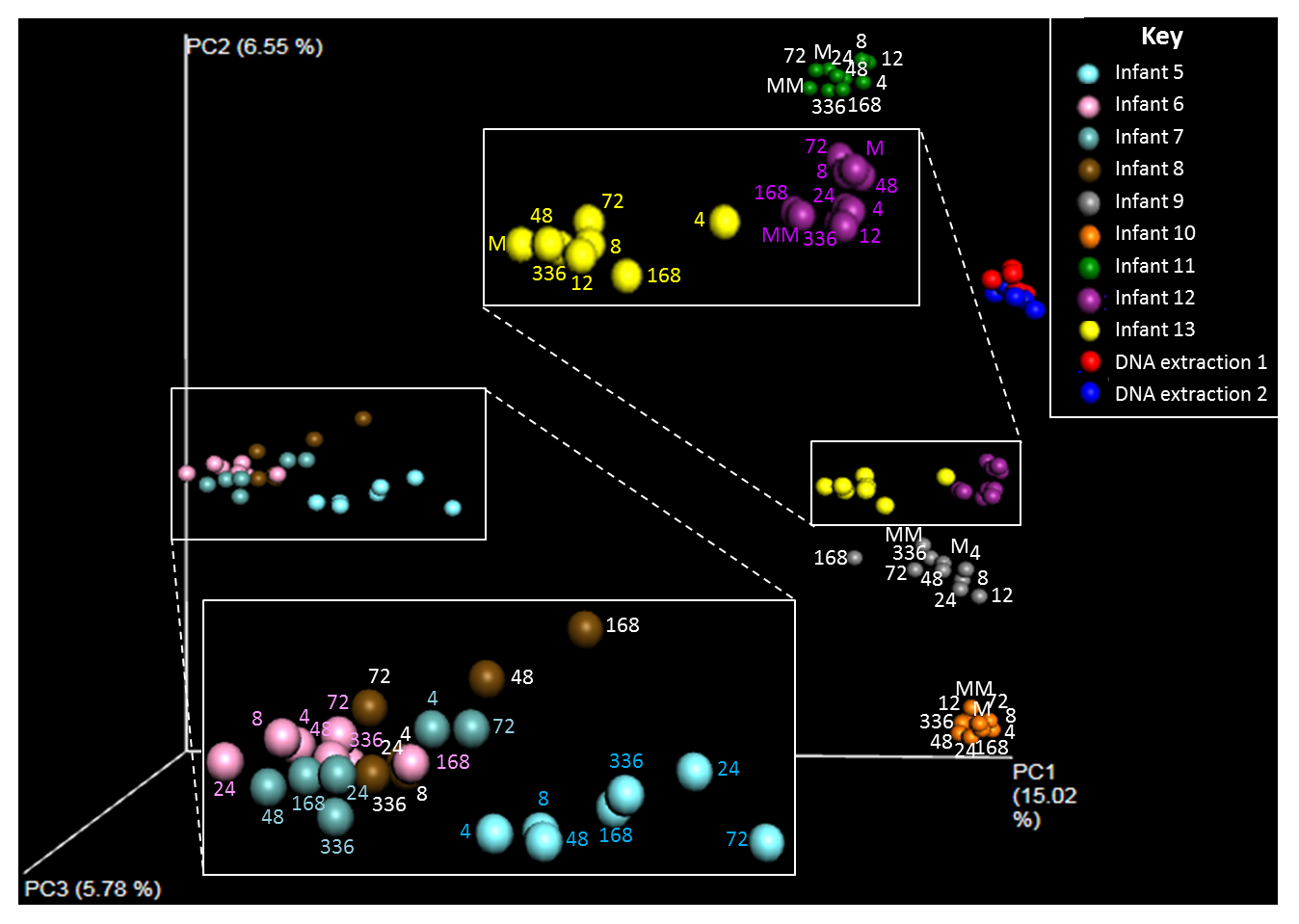
*

*Figure S8 – PCoA plot using the Jaccard dissimilarity metric. Samples labelling as Supplementary Figure 5.*
