# Supplementary material for: Latitude in sample handling and storage for infant faecal microbiota studies: the elephant in the room?
Source: Microbiome. 2016 Jul 30;4:40. doi: 10.1186/s40168-016-0186-x (PMC4967342; doi:10.1186/s40168-016-0186-x)
Supplement: Additional file 8: Figure S9. — The microbial communities of the samples used in the room temperature storage experiment, summarised to phyla. From top left to bottom right, infants 5 to 13, with infants 5–8 being premature (simple faecal communities) and 9–13 being term (more complex faecal communities). (DOCX 154 kb) [file 40168_2016_186_MOESM8_ESM.docx]

**Additional file 8: Figure S9**


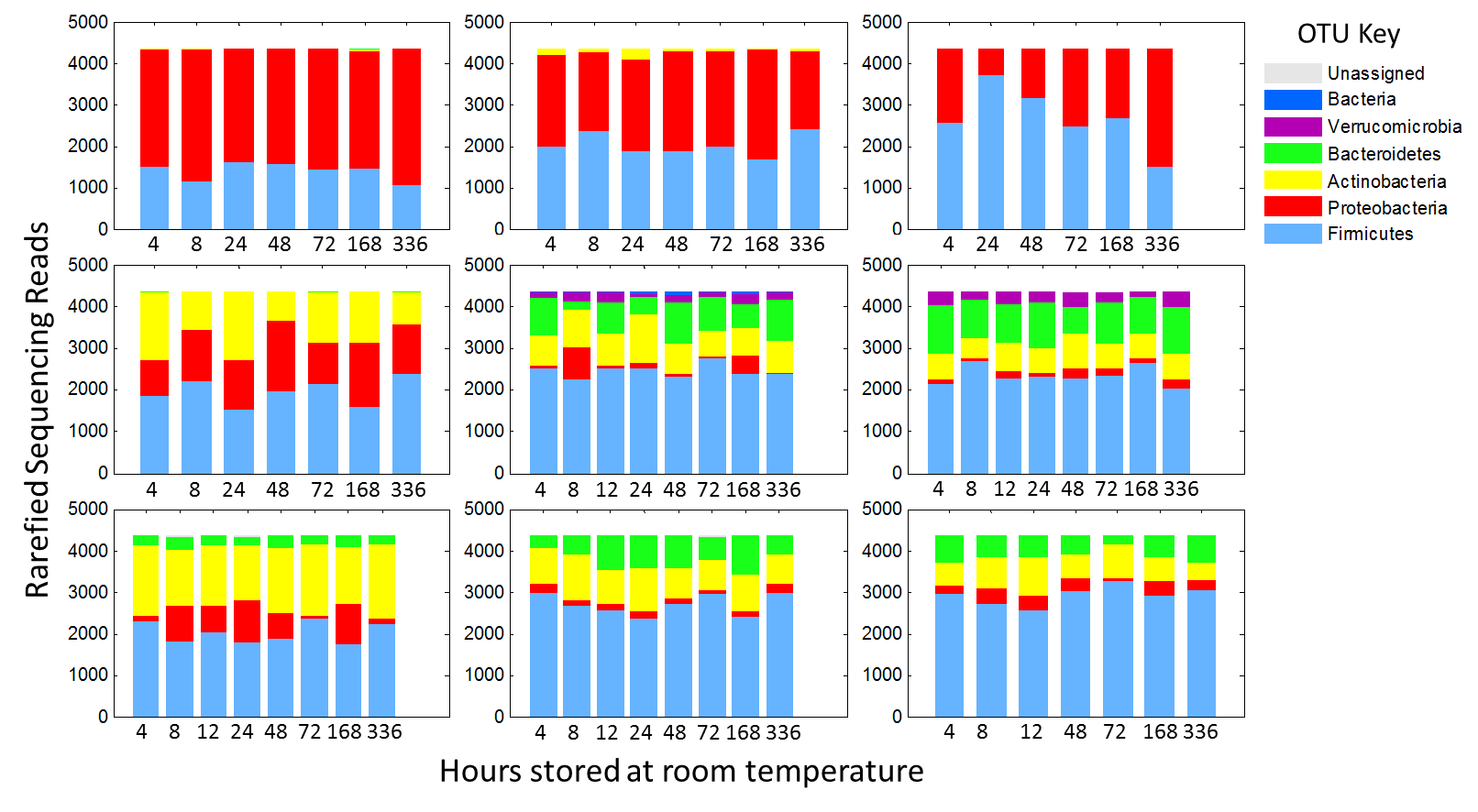


*Additional file 8: Figure S9 - The microbial communities of the samples used in the room temperature storage experiment, summarised to phyla. From top left to bottom right, infants 5 to 13, with infants 5-8 being premature (simple faecal communities) and 9-13 being term (more complex faecal communities).*
