# Supplementary material for: Latitude in sample handling and storage for infant faecal microbiota studies: the elephant in the room?
Source: Microbiome. 2016 Jul 30;4:40. doi: 10.1186/s40168-016-0186-x (PMC4967342; doi:10.1186/s40168-016-0186-x)
Supplement: Additional file 9: Figure S10. — The microbial communities of the samples used in the mail experiment. (DOCX 97 kb) [file 40168_2016_186_MOESM9_ESM.docx]

**Additional file 9: Figure S10**


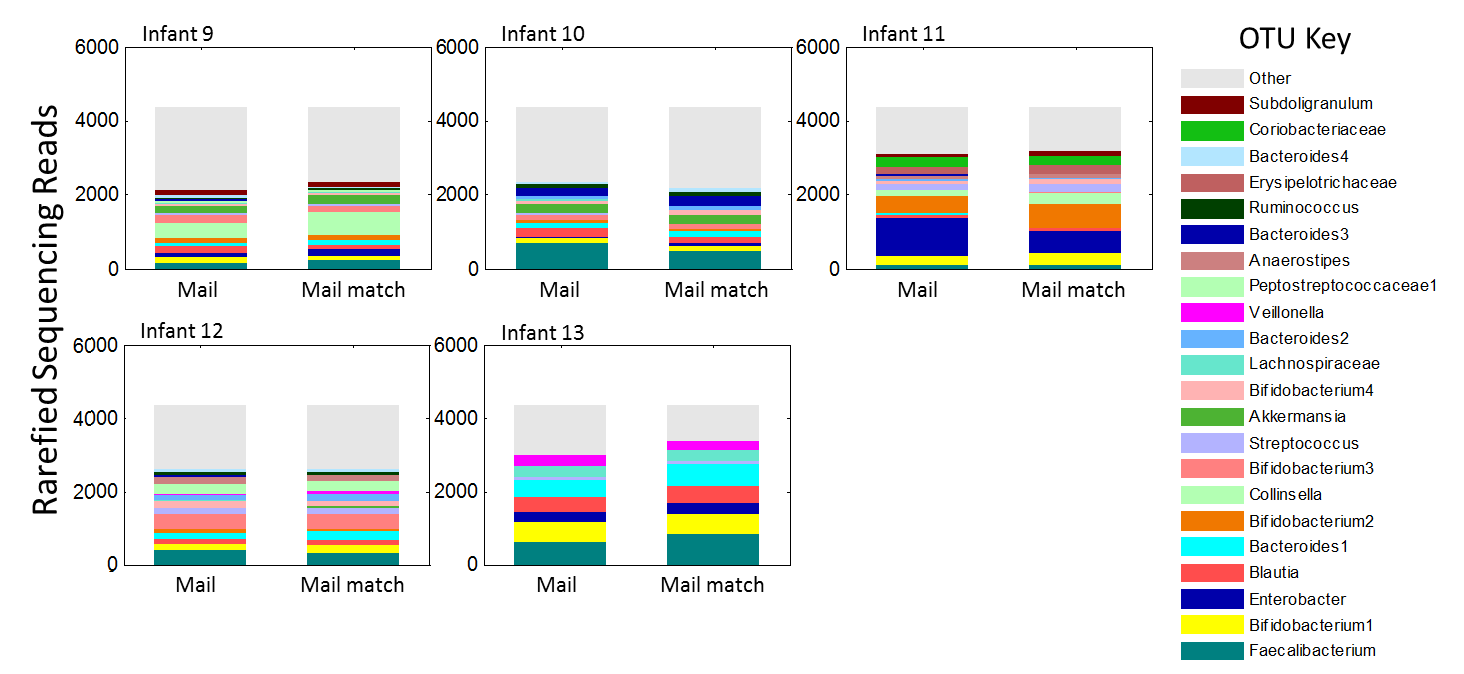


*Additional file 9: Figure S10 - The microbial communities of the samples used in the mail experiment.*
